# The urgent need for Middle East respiratory syndrome coronavirus surveillance in Somalia

**DOI:** 10.1016/j.ijregi.2025.100806

**Published:** 2025-11-16

**Authors:** Kassim Abdi Jimale, Shafie Abdulkadir Hassan, Abdifetah Ibrahim Omar

**Affiliations:** Faculty of Medicine and Health Sciences, Jamhuriya University of Science and Technology, Mogadishu, Somalia

## Abstract

• Somalia lacks MERS-CoV surveillance despite its large camel population.
• Camel trade with the Arabian Peninsula heightens cross-border transmission risk.
• Urgent One Health surveillance is needed to close this critical data gap.

Dear editor,

We read with great interest the recent editorial by Al-Tawfiq [1] on the global epidemiology and public health challenges of Middle East respiratory syndrome coronavirus (MERS-CoV). The author rightly highlights the central role of robust surveillance in managing this persistent zoonotic threat. However, the global surveillance map described, which is based on reported data, contains a significant and dangerous blind spot in Somalia.

The review underscores the intrinsic link between MERS-CoV and dromedary camels [2,3]. This connection makes the complete absence of surveillance data from Somalia, a nation possessing the world's largest dromedary camel population, particularly alarming (Table 1) [4]. This high-risk ecological setting is compounded by extensive socio-economic factors [5]. As Africa's largest livestock exporter, Somalia’s robust trade with the MERS-CoV-endemic Arabian Peninsula creates a persistent conduit for cross-border virus transmission. Consequently, infections among high-risk Somali populations, such as herders and traders, could easily go undetected or misdiagnosed, undermining the global control efforts Al-Tawfiq [1] advocates for.

**Table 1:** Zonal distribution of dromedary camel population in Somalia.

Livelihood zone	Camel population ('000 heads)	As % of zone's total livestock	As % of Somalia's total camel population
North-Western	1,308.26	10%	20%
North-Eastern	1,347.70	10%	21%
Central	1,003.34	16%	16%
Southern	1,217.47	23.8%	19.3%
Juba Valley	1,417.46	22.6%	22.5%

Source: Data extracted from the Livestock Population Distribution Map, FSAU/FAO, 1999.

The robust livestock trade, with projections nearing 4 million for 2025, creates a persistent conduit for the cross-border transmission of the virus, potentially re-seeding outbreaks in destination countries [6]. This epidemiological silence is not evidence of absence. Studies in neighboring Kenya and Ethiopia have confirmed high rates of MERS-CoV circulation in their camel herds. The lack of similar seroprevalence or molecular data from Somalia represents a critical knowledge gap that jeopardizes both national and global health security [7,8]. This epidemiological silence is not evidence of absence; rather, it is a critical blind spot that undermines both national and global health security.

## Future perspectives

We strongly support Al-Tawfiq [1]’s call for enhanced surveillance; however, we urge that this call be directed specifically toward this high-risk, data-poor region. We call upon national health authorities, with support from international partners like the World Health Organization and FAO, to implement a robust “One Health” surveillance strategy in Somalia. This must include systematic surveillance in dromedary camels at key markets and export points, targeted surveillance of high-risk human populations, and strengthening in-country laboratory diagnostic capacity. Addressing the MERS-CoV question in Somalia is not merely an academic exercise but a crucial, time-sensitive imperative for preventing the future outbreaks highlighted in the editorial.

## Funding

This work did not receive any grant from funding agencies in the public, commercial, or not-for-profit sectors.

## Author contributions

K.A.J: conceptualized and designed the study, conducted the literature review. S.A.H and A.I.O: contributed to the manuscript, wrote the first draft and critically revised the manuscript for important intellectual content. All authors approved the final draft before submission.

## Declarations of competing interest

The authors have no competing interests to declare.
